# Different patterns of inflammatory and angiogenic factors are associated with peritoneal small solute transport and peritoneal protein clearance in peritoneal dialysis patients

**DOI:** 10.1186/s12882-018-0921-6

**Published:** 2018-05-23

**Authors:** Yuanyuan Shi, Hao Yan, Jiangzi Yuan, He Zhang, Jiaying Huang, Zhaohui Ni, Jiaqi Qian, Wei Fang

**Affiliations:** Department of Nephrology, Renji Hospital, School of Medicine, Shanghai Jiao Tong University, No.1630, Dongfang Road, Pudong New Area, Shanghai, 200127 China

**Keywords:** Angiogenic factors, Inflammatory factors, Peritoneal small solute transport, Peritoneal protein clearance, Peritoneal dialysis

## Abstract

**Background:**

Both peritoneal small solute transport and peritoneal protein clearance are closely linked to outcomes in peritoneal dialysis (PD) patients. However, the associated factors of these two components are not fully understood so far. This study aimed to investigate the association between a panel of systemic and peritoneal inflammatory and angiogenic factors and peritoneal solute transport properties.

**Methods:**

Stable PD patients in PD center of Renji Hospital, School of Medicine, Shanghai Jiao Tong University were enrolled in present study. Serum and overnight effluent markers including angiopoietin-1 (Ang-1), angiopoietin-2 (Ang-2), sTie-2, VEGF, IL-6 and IL-10 were determined. Mass transfer area coefficient of creatinine (MTACcr) and peritoneal protein clearance (Prcl) were calculated. Multivariable linear regression was used to examine the association between these markers and MTACcr as well as Prcl.

**Results:**

A total of 320 patients were enrolled in present study, which consisted of 166 (51.9%) males with a mean age of 56.8 ± 14.2 years and a median PD duration of 32.5 (9.0–56.3) months. Multiple regression analyses showed that BSA, history glucose exposure, dialysate IL-6 AR and dialysate Ang-1 AR were independent associated factors of MTACcr, while BSA and serum Ang-1 were independent associated factors of Prcl.

**Conclusions:**

MTACcr representing peritoneal small-solute transport and Prcl representing peritoneal large molecular transport are associated with slightly different panels of inflammatory and angiogenic factors.

## Background

Peritoneal dialysis (PD) is a well-established renal replacement therapy for patients with end stage renal disease (ESRD) [1]. During long-term PD treatment, however, the peritoneal membrane undergoes functional as well as structural alterations, which may be the consequence of many causes such as peritonitis and continuous exposure to bioincompatible dialysis solutions with high concentrations of glucose and glucose degradation products, low pH as well as high osmolality etc. [2].

The most common functional alteration during long-term PD is increased peritoneal small-solute transport rate measured by dialysate-to-plasma (D/P) ratios or mass transfer area coefficients (MTAC) [3, 4], which is the major contributor to impaired ultrafiltration and ultimately discontinuation of treatment [5]. It has been well appreciated that high D/Pcr represented an independent risk factor for mortality and technique failure in PD patients [6–8] as a consequence of increased glucose absorption from peritoneal cavity and inadequate dialysis, leading to suboptimal ultrafiltration capacities, fluid overload and malnutrition [9]. On the other hand, patients may lose approximately 5 g total protein or 4 g albumin daily from the peritoneal cavity during PD therapy [10]. Accumulating studies have suggested that peritoneal protein clearance (PrCl) was linked to poor outcomes in PD patients. These studies postulated that Prcl was associated with a higher prevalence of peripheral arterial disease [11], cardiovascular events [12], peritonitis [13], and increased mortality in PD patients [14]. So both peritoneal small solute transport and peritoneal protein clearance are closely linked to outcomes in PD patients.

Angiopoietin-1 (Ang-1) and angiopoietin-2 (Ang-2) are antagonistic ligands of the TEK tyrosine kinase-2 (Tie-2) receptor [15]. It has been shown that Ang-1 was a strong anti-permeability factor that decreased vascular permeability and protected the adult vasculature against plasma leakage [16–18]. Experimental overexpression of Ang-1 during sepsis could reduce mortality through preservation of vessel integrity [19]. In contrast, increased circulating Ang-2 has been reported in various diseases with endothelial activation [20–22]. Additionally, vascular endothelial growth factor (VEGF) might inhibit vascular stabilization in an angiopoietin- dependent manner [23].

Despite both peritoneal small solute transport and peritoneal protein clearance were closely linked to outcomes in PD patients, the associated factors of these two components still remained unclear. Therefore, this study was designed to investigate the possible associated factors of peritoneal small-solute and large-molecular transport in patients undergoing PD.

## Methods

### Ethics statement

The study protocol was approved by the Ethics Committee of Renji Hospital, School of Medicine, Shanghai Jiao Tong University, China (number: [2013] N022; year: January/2014). Written consent was given by the patients for their information to be stored in the hospital database and used for research.

### Patient population

All clinically stable CAPD patients during April 1, 2014 to May 31, 2015 in PD center of Renji Hospital, School of Medicine, Shanghai Jiao Tong University were included in present study. Exclusion criteria included: (1) presence of systemic inflammatory disease, peritonitis or fluid overload within 3 months prior to the study; (2) malignancy; (3) taking glucocorticoid or immunosuppressive agents during the past 1 year, and (4) acute cardiocerebrovascular events that occurred within 3 months prior to the study. All patients were dialyzed with glucose-based peritoneal dialysis fluid (Dianeal®, Baxter). All enrolled patients were asked to come to PD center to take a standard peritoneal equilibration test. Our PD nurses performed the PET and obtained blood and effluent samples strictly according to the study procedure, so there was no missing data.

### Protocol

The following demographic characteristics were collected at the enrollment of study: age, gender, height, weight, underlying cause of ESRD, date of PD initiation, previous peritonitis episodes, taking Angiotensin-Converting Enzyme Inhibitor/Angiotensin Receptor Blocker (ACEI/ARB) or not, presence of comorbid diseases such as diabetes mellitus (DM) and cardiovascular disease (CVD). CVD was defined as a previous history of coronary artery disease, peripheral vascular disease or cerebrovascular disease. The historical dialysis regimen was collected to calculate the amount of historical glucose exposure according to Davies et al. [24].

On the night prior to PD center visit, the patient was asked to perform a dialysis exchange using his or her usual overnight dialysis regimen, and the overnight effluent was fully drained the next morning in the PD center. We weighed the bag of drained effluent to assess the volume, and the dwell duration was recorded. A 10-ml sample was collected from the drained effluent, which was immediately stored at − 70 °C for determination of the concentrations of cytokines. A standard peritoneal equilibration test (PET) [25] was initiated for each enrolled patient. A whole blood sample was collected at 120 min and the serum was separated and stored at − 70 °C. Residual renal function (RRF) was assessed as the average of urea and creatinine clearance from a 24-h urine collection. Urea clearance index (Kt/V urea) was derived from the collection of the 24-h PD effluent and 24-h urine. We also measured laboratory parameters of each patient including hemoglobin, serum albumin, and high-sensitivity C-reactive protein (hs-CRP).

### Evaluation of peritoneal small-solute transport rate and peritoneal protein clearance

Mass transfer area coefficient for creatinine (MTACcr) was calculated using the simplified Garred equation [26]: MTAC (ml/min) = (Vd/t) ln ({Vi · P} / {Vd [P – Dt]}), where Vd is the drained volume, t is the dwell time (240 min), Vi is the instilled dialysate volume, P is the plasma concentration, and Dt is the dialysate concentration at the end of dwell time, normally determined in the dialysate after drainage. The 4-h ultrafiltration (4 h UF) during PET and 24-h ultrafiltration of each patient were assessed.

Peritoneal dialysate protein losses were calculated from the collection of 24-h peritoneal dialysate effluent by the Biuret method. A validated correction factor was used for the calculation of Prcl [10]: 24-h dialysate protein loss/(serum albumin/0.4783). Prcl was expressed as ml of plasma cleared per day.

### Assessment of markers of inflammation and angiogenesis

The concentrations of inflammatory and angiogenic makers in the serum and overnight effluent were determined using the ELISA technique. All samples were run simultaneously for each mediator to avoid intra- and inter-assay variations. Ang-1, Ang2, sTie-2 and VEGF levels were measured using commercially available Quantikine ® ELISA kits (R&D Systems Inc., Minneapolis, MN, USA) according to the manufacturer’s instructions. IL-6 and IL-10 concentrations were determined by Human IL-6 ELISA Ready-SET-Go! (eBioscience) and Human IL-10 ELISA Ready-SET-Go! (eBioscience) respectively. Due to the concentrations of dialysate cytokines were influenced by ultrafiltration volume which was affected by peritoneal solute transport rate and dwell time, the dialysate appearance rate (AR) was calculated as dialysate concentration times the drained volume divided by the dwell time and expressed as picograms or nanograms per minute.

### Statistical analyses

Data were subjected to the Kolmogorov-Smirnov test to examine their distribution. Continuous variables were presented as mean ± SD or median and inter quartile range (IQR), depending on their distribution. Categorical data were expressed as proportions. Correlations between variables were assessed by the Pearson’s or Spearman rank correlation analyses depending on data distribution. Multivariate regression analysis was used to assess the predictors for MTACcr and Prcl (dependent variable), respectively. Log (base 10)- transfer was applied to non-normally distributed values before entering regression analysis. Statistical significance was accepted at a two-sided *P*-value of < 0.05. The analyses were performed using IBM SPSS Statistics software version 21.0.

## Results

### Patient characteristics

A total of 320 patients were enrolled in present study. Among them, 166 (51.9%) were males with a mean age of 56.8 ± 14.2 years and a median PD duration of 32.5 (9.0–56.3) months. 79(24.7%) patients had diabetes mellitus as comorbidity. The demographic and clinical characteristics of the study are described in Table 1.

**Table 1:** Demographic and clinical characteristics of the study subjects (*n* = 320)

Variable	Value
Age (years)	56.8 ± 14.2
Gender (Male)	166 (51.9%)
BSA (m^2^)	1.62 ± 0.17
Systolic pressure (mmHg)	139 ± 21
Diastolic pressure (mmHg)	87 ± 13
PD vintage (months)	32.5 (9.0–56.3)
Underlying renal disease	
Chronic glomerulonephritis [n (%)]	165 (51.6%)
Diabetic nephropathy [n (%)]	44 (13.6%)
Hypertension [n (%)]	23 (7.2%)
Polycystic kidney disease [n (%)]	10 (3.1%)
Others [n (%)]	49 (15.4%)
Unknown [n (%)]	29 (9.1%)
Comorbidity	
Diabetes mellitus [n (%)]	79 (24.7%)
Cardiovascular disease [n (%)]	78 (24.4%)
Number of patients who had peritonitis [n (%)]	80 (25%)
ACEI/ARB taking [n (%)]	188 (58.8%)
Historical glucose exposure (g)	117,720 (37420–249,320)
Hemoglobin (g/L)	107.1 ± 17.2
Serum albumin (g/L)	37.0 ± 4.7
hs-CRP (mg/L)	2.5 (0.9–6.3)
Dialysis adequacy	
Total Kt/V urea	1.96 ± 0.38
Peritoneal Kt/V urea	1.56 ± 0.36
Renal Kt/V urea	0.24 (0–0.65)
RRF (ml/min)	0.96 (0–2.88)
nPCR (g/Kg/day)	0.88 ± 0.19
D/P cr at 4 h	0.62 ± 0.11
MTACcr (ml/min)	7.64 (5.97–9.56)
Prcl (ml/d)	69.70 (52.31–90.23)

Values are expressed as mean ± SD or median (IQR) for continuous data and number of patients (percent) for categorical data. *BSA* body surface area, *ACEI/ARB* Angiotensin-Converting Enzyme Inhibitor/Angiotensin Receptor Blocker, *hs-CRP* high sensitivity C-reactive protein, *D/P cr* dialysate/plasma creatinine ratio, *RRF* residual glomerular filtration rate, *nPCR* normalized protein catabolic rate. *MTACcr* Mass transfer area coefficient for creatinine, *Prcl* Peritoneal dialysate protein losses

### Correlation between clinical & biochemical parameters and peritoneal transport characteristics

Significant positive correlations were seen between MTACcr and BSA (*r* = 0.25, *p* < 0.001), systolic blood pressure (*r* = 0.153, *p* = 0.007), PD vintage (*r* = 0.162, *p* = 0.003) as well as historical glucose exposure (*r* = 0.121, *p* = 0.030). In contrast, significant negative correlations were found between MTACcr and serum albumin (*r* = − 0.232, *p* < 0.001), 24-h ultrafiltration (r = − 0.2 *p* < 0.001,), as well as 4-h ultrafiltration (*r* = − 0.317, *p* < 0.001). Higher Prcl was seen with advanced age (*r* = 0.226, *P* < 0.001), increasing BSA (*r* = 0.251, *p* < 0.001), higher systolic blood pressure (*r* = 0.276, *P* < 0.001), higher RRF (*r* = 0.409, *p* < 0.001) and more 24-h urine volume (*r* = 0.405, *p* < 0.001). The presence of diabetes mellitus and cardiovascular disease were also significantly associated with higher Prcl (*r* = 0.187, *P* = 0.002 and *r* = 0.188, *P* = 0.002, respectively). Significant negative correlation was existed between Prcl and serum albumin (*r* = − 0.497, *p* < 0.001), nPCR (*r* = − 0.207, *p* = 0.001) and 4 h-ultrafiltration (*r* = − 0.133, *p* = 0.030).

### Correlation between systemic & dialysate markers and peritoneal transport characteristics

A significant positive correlation was found between MTACcr and peritoneal protein clearance (Prcl) (Spearman’s rho = 0.326, *P* < 0.001). As shown in Tables 2 and 3, in the overnight effluent, Ang-1AR (*r* = − 0.201, *P* < 0.001) was inversely correlated with MTACcr, while IL-6 AR (*r* = 0.190, *P* = 0.002) was positively correlated with MTACcr. Ang-2 AR (Spearman’s rho = 0.123, *P* = 0.028) and sTie-2 AR (Spearman’s rho = 0.142, *P* = 0.011) were positively correlated with Prcl, respectively. Serum Ang-1 concentration was inversely correlated with both MTACcr (*r* = − 0.205, *p* < 0.001) and Prcl (Spearman’s rho = − 0.203, *P* < 0.001). Serum IL-6 concentration was positively correlated with both MTACcr (*r* = 0.173, *p* = 0.004) and Prcl (Spearman’s rho = 0.136, *p* = 0.016).

**Table 2:** Correlation between serum markers and peritoneal transport characteristics

		Log Ang-1	Log Ang-2	Log sTie-2	Log IL-6	Log IL-10	Log VEGF
Log MTACcr	r	− 0.205	0.101	−0.054	0.173	−0.021	− 0.105
	p	< 0.001	0.076	0.337	0.004	0.709	0.064
Log Prcl	rho	− 0.203	0.046	−0.006	0.136	−0.047	0.001
	p	< 0.001	0.408	0.919	0.016	0.407	0.990

**Table 3:** Correlation between effluent markers and peritoneal transport characteristics

		LogAng-1 AR	LogAng-2 AR	Log sTie-2 AR	Log IL-6 AR	Log IL-10 AR	Log VEGF AR
LogMTACcr	r	−0.201	− 0.054	− 0.021	0.167	− 0.094	− 0.053
	p	< 0.001	0.342	0.704	0.003	0.097	0.351
LogPrcl	rho	0.032	0.123	0.142	0.093	0.097	0.034
	p	0.574	0.028	0.011	0.10	0.085	0.542

### Correlation between systemic and dialysate markers

**Fig. 1:**
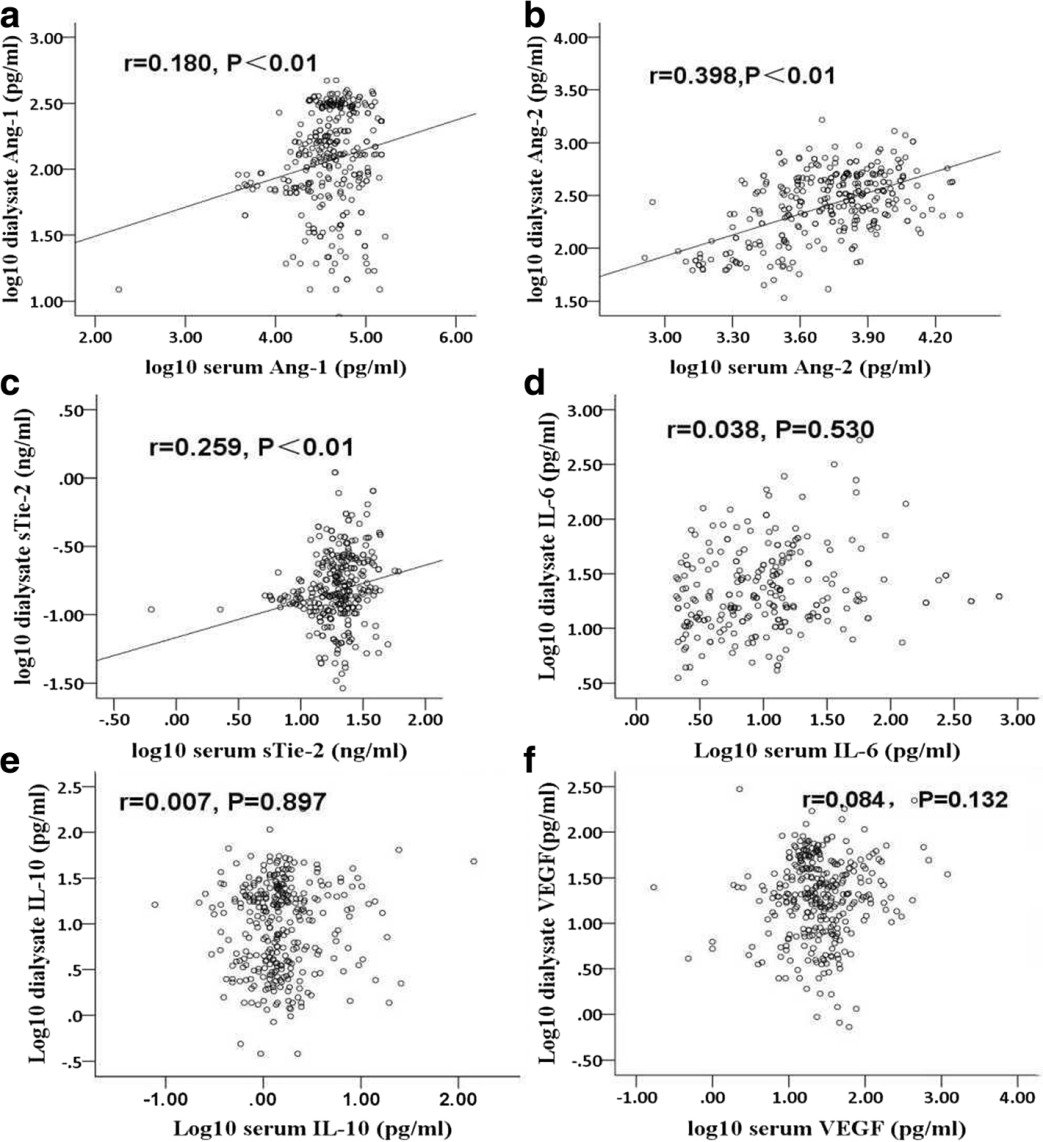
Correlation between systemic and dialysate levels of Ang-1 (**a**), Ang-2 (**b**), sTie-2 (**c**), IL-6 (**d**), IL-10 (**e**) and VEGF (**f**), respectively

Serum Ang-1, Ang-2 and sTie-2 levels in this cohort were 42,015.00 (27,810.00–63,320.00) pg/ml, 5451.43 (3429.99–7740.90) pg/ml, and 20.13 (15.36–25.49) ng/ml, respectively. Serum IL-6, IL-10 and VEGF concentrations were 5.56 (1.88–13.62) pg/ml, 1.38 (0.99–2.02) pg/ml, 26.94 (14.42–46.70) pg/ml, respectively. Ang-1, Ang-2, sTie-2, IL-6, IL-10 and VEGF levels in PDF in this cohort were 130.09 (70.42–266.78) pg/ml, 300.14 (170.69–464.93) pg/ml, 0.15 (0.11–0.24) ng/ml, 17.79 (10.35–33.81) pg/ml, 1.48 (0.79–2.74) pg/ml, and 28.03 (18.26–49.18) pg/ml, respectively. The serum to dialysate concentration ratios of Ang-1, Ang-2, sTie-2, IL-6, IL-10 and VEGF are 269.44, 18.97, 119.92, 0.29, 0.93 and 0.95, respectively. There was a significant positive correlation between the systemic and dialysate concentrations of Ang-1 (*r* = 0.180, *P* < 0.01), Ang-2 (*r* = 0.398, *P* < 0.01) and sTie-2 (*r* = 0.259, *P* < 0.01), respectively. However, no correlations of IL-6 (*r* = 0.038, *P* = 0.530), IL-10 (*r* = 0.007, *P* = 0.897) and VEGF (*r* = 0.084, *P* = 0.132) were found between their systemic and dialysate concentrations (Fig. 1).

### Independent associated factors of MTACcr and Prcl

To analyze the independent contributions of various cytokines to the variance of MTACcr and Prcl, we performed a series of multiple regression models. After adjustment for age, sex, diabetes mellitus, BSA, PD vintage, history glucose exposure, nPCR and RRF, serum IL-6 [β = 0.105 (0.011–0.197), *P* = 0.028] and serum Ang-1 [β = − 0.122 (− 0.217– -0.026), *P* = 0.012] were independently associated with MTACcr in the Model2; dialysate IL-6 AR [β = 0.130 (0.061–0.199), P = 0.01] and dialysate Ang-1 AR [β = − 0.082 (− 0.157– -0.007), *P* = 0.032] were independently associated with MTACcr in the Model3 (Table 4). However, after adjustment for age, sex, diabetes mellitus, BSA, PD vintage, history glucose exposure, nPCR and RRF, serum IL-6 [β = 0.048 (0.019–0.077), *P* = 0.001] and serum Ang-1 [β = − 0.068 (− 0.127,-0.010), *P* = 0.003] were independently associated with Prcl. (Table 5). When including both serum and dialysate markers in a single model (Model4), BSA [β = 0.0243 (0.0114,0.0371), *P* < 0.01], history glucose exposure [β = 0.055 (0.020, 0.140), *P* < 0.01], dialysate IL-6 AR [β = 0.083 (0.027, 0.139), *P* < 0.01] and dialysate Ang-1 AR [β = − 0.069 (− 0.118, − 0.019), *P* < 0.01] were independent associated factors of MTACcr, however, only BSA[β = 0.058 (0.036–0.079), *P* < 0.01] and serum Ang-1 [β = − 0.040 (− 0.067, − 0.013), *P* < 0.01] were independent associated factors of Prcl.

**Table 4:** Multiple regression models for MTACcr (ml/min) in PD patients

	Model 1 (β, 95% CI) (*r*^2^ = 0.35)	Model 2 (β, 95% CI) (*r*^2^ = 0.39)	Model 3 (β, 95% CI) (*r*^2^ = 0.47)	Model 4 (β, 95% CI) (*r*^2^ = 0.54)
Age (years)	0.013 (−0.208–0.042)	0.005 (− 0.001–0.018)	0.006 (− 0.005–0.004)	0.002 (− 0.005–0.003)
Sex (males %)	0.054 (− 0.408–0.517)	0.014 (− 0.099–0.128)	0.015 (− 0.046–0.118)	0.006 (− 0.092–0.265)
DM	−0.026 (−1.060–0.008)	−0.028 (− 0.062–0.006)	−0.093 (− 0.177–0.012)	− 0.033 (− 1.535–0.879)
BSA (m^2^)	0.094 (0.042–0.145)	0.052 (0.019–0.085)	0.082 (0.031–0.132)	0.0243 (0.0114, 0.0371)
PD vintage(months)	0.042 (− 0.148–0.157)	0.004 (− 0.036–0.043)	0.003 (− 0.037–0.042)	0.007 (− 0.003–0.027)
Historical glucose exposure(g)	0.110 (0.073–0.349)	0.095 (0.027–0.531)	0.0861 (0.024–0.420)	0.055 (0.020, 0.140)
nPCR(g/Kg/day)	−0.034 (− 0.118–0.051)	−0.106 (− 0.408–0.028)	−0.107 (− 0.401–0.909)	−0.051 (− 0.449–0.972)
RRF(ml/min)	0.006 (− 0.006–0.018)	0.146 (− 0.276–0.567)	0.093 (− 0.305–0.492)	0.069 (− 0.167–0.443)
Log serum IL-6		0.105 (0.011–0.197)		0.001 (− 0.006–0.008)
Log serum IL-10		− 0.004 (− 0.025–0.018)		0.0013 (− 0.013–0.051)
Log serum VEGF		0.018 (− 0.010–0.045)		0.003 (− 0.005–0.011)
Log serum Ang-1		− 0.122 (− 0.217– -0.026)		− 0.005 (− 0.075–0.045)
Log serum Ang-2		0.032 (− 0.024–0.089)		− 0.005 (− 0.064–0.051)
Log serum sTie-2		0.030 (− 0.026–0.086)		− 0.004 (− 0.008–0.005)
Log dialysate IL-6 AR			0.130 (0.061–0.199)	0.083 (0.027, 0.139)
Log dialysate IL-10 AR			−0.112 (− 0.187–0.036)	0.001 (− 0.037–0.013)
Log dialysate VEGF AR			0.092 (− 0.005–0.188)	−0.001 (− 0.021–0.018)
Log dialysate Ang-1 AR			−0.082 (− 0.157– -0.007)	−0.069 (− 0.118, − 0.019)
Log dialysate Ang-2 AR			0.013 (− 0.002–0.028)	0.002 (− 0.003–0.003)
Log dialysate sTie-2 AR			0.086 (− 0.006–0.178)	0.003 (− 0.008–0.005)

**Table 5:** Multiple regression models for Prcl (ml/d) in PD patients

	Model 1 (β, 95% CI) (*r*^2^ = 0.37)	Model 2 (β, 95% CI) (*r*^2^ = 0.59)	Model 3 (β, 95% CI) (*r*^2^ = 0.38)	Model 4 (β, 95% CI) (*r*^2^ = 0.48)
Age (years)	0.035 (−0.035–0.105)	0.022 (− 0.046–0.090)	0.016 (− 0.042–0.074)	0.005 (−0.006–0.016)
Sex (males %)	− 0.052 (− 0.121–0.017)	−0.065 (− 0.330–0.199)	−0.029 (− 0.087–0.030)	−0.001 (− 0.017–0.043)
DM	0.040 (0.022–0.058)	0.016 (− 0.034–0.066)	0.0171 (− 0.01–0.044)	0.007 (−0.015–0.032)
BSA (m^2^)	0.217 (0.044–0.429)	0.156 (0.052–0.482)	0.171 (0.041–0.506)	0.058 (0.036–0.079)
PD vintage (months)	0.103 (− 0.106–0.413)	0.053 (− 0.017–0.123)	0.091 (−0.067–0.086)	0.002 (− 0.001–0.007)
Historical glucose exposure (g)	0.041 (− 0.035–0.102)	0.008 (− 0.042–0.070)	0.006 (−0.047–0.120)	0.001 (− 0.007–0.003)
nPCR (g/Kg/day)	−0.177 (− 0.346– -0.001)	−0.105 (− 0.047–0.070)	− 0.105 (− 0.035–0.002)	−0.103 (− 0.357–0.139)
RRF (ml/min)	0.041 (− 0.030–0.113)	0.019 (− 0.052–0.070)	0.017 (−0.054–0.078)	0.003 (− 0.005–0.011)
Log serum IL-6		0.048 (0.019–0.077)		0.004 (− 0.050–0.059)
Log serum IL-10		−0.008 (− 0.010–0.044)		−0.003 (− 0.009–0.032)
Log serum VEGF		0.005 (−0.025–0.018)		−0.002 (− 0.008–0.004)
Log serum Ang-1		−0.068 (− 0.127, –0.010)		−0.040 (− 0.067, –0.013)
Log serum Ang-2		0.016 (−0.034–0.066)		0.001 (− 0.001–0.002)
Log serum sTie-2		− 0.001 (− 0.026–0.086)		−0.0002 (− 0.002–0.001)
Log dialysate IL-6 AR			0.086 (− 0.006–0.188)	0.004 (− 0.005–0.009)
Log dialysate IL-10 AR			−0.046 (− 0.077–0.013)	−0.002 (− 0.008–0.004
Log dialysate VEGF AR			−0.020 (− 0.046–0.005)	−0.009 (− 0.012–0.001
Log dialysate Ang-1 AR			0.100 (− 0.013–0.067)	0.009 (− 0.004–0.042)
Log dialysate Ang-2 AR			0.036 (− 0.001–0.072)	0.004 (− 0.008–0.00
Log dialysate sTie-2 AR			0.025 (− 0.014–0.064)	−0.002 (− 0.008–0.004)

## Discussion

Our results showed that BSA, history glucose exposure, dialysate IL-6 AR and dialysate Ang-1 AR were independent associated factors of MTACcr, while BSA and serum Ang-1 were independently associated with Prcl, suggesting that peritoneal small-solute transport and peritoneal large molecular transport may be affected by slightly different panels of inflammatory and angiogenic factors.

During long-term PD, the peritoneal membrane undergoes both structural and functional alterations [2]. The structural changes include denudation of the mesothelial cell layer, thickening of the sub-mesothelial space, neoangiogenesis, and thickening of the vascular wall by type IV collagen deposition [27]. In parallel with these alterations, the most common functional alteration during long-term PD is increased peritoneal small-solute transport rate [3, 4], which may impair ultrafiltration capacity [5]. In our previous study, we reported that MTACcr increased significantly over time while ultrafiltration capacity decreased [28]. High peritoneal small-solute transport rate has been recognized to be an independent risk factor for mortality and technique failure in PD patients [6–8]. A meta-analysis including 6648 PD patients showed that a higher peritoneal small-solute transport rate was associated with a significantly higher mortality and a trend of higher technique failure: 19 studies were pooled to generate a summary mortality relative risk of 1.15 for every 0.1 increase in the D/Pcr [7]. These studies suggested the close association of increased peritoneal small-solute transport with poor outcomes in PD patients.

Peritoneal protein clearance (Prcl) in PD patients mainly reflects protein leakage across the large pores, which is equivalent to large-pore flow (JvL). It has been shown that patients starting PD with active CVD had higher protein and albumin levels in their peritoneal effluent, and cardiovascular events were more frequent in patients with greater peritoneal albumin losses [12]. Heaf et al. [29] found that JvL was related to hypoalbuminemia and mortality in PD patients. Dong et al. [13] reported recently that baseline peritoneal protein leakage was an independent predictor of peritonitis even after adjustment of systemic inflammation state. Perl et al. [14] reported that increased PrCl at the start of PD was a predictor of death independent of baseline small solute transport status and other important characteristics. All these studies suggested that PrCl also had a close association with poor outcomes in PD patients.

Since it has been well appreciated that many cytokines interfere with the permeability of various biological membranes, it is likely that they may also be involved in the regulation of peritoneal permeability. Ang-1, continuously produced and released by pericytes and vascular smooth muscle cells, could maintain structure integrity of vasculature and protect the endothelium from excessive activation by cytokines and growth factors [30]. Ang-1 could inhibit vascular permeability in response to thrombin and VEGF in vitro [16, 17]. It has been reported that Ang-1 maintained the integrity of endothelial monolayers in mature animals and protected the adult vasculature against plasma leakage [18]. On the other hand, increased Ang-2 expression in endothelial cells has been observed with stimulation of high glucose [31] or tumor necrosis factor-α [32], both frequently observed in PD patients. A balanced Ang-1/Ang-2 ratio is required for maintaining the normal function of the endothelial layer [15]. It has been shown [33] in atherosclerotic plaques with high microvessel density that the local balance between Ang-1 and Ang-2 was in favor of Ang-2. In present study, we found that dialysate Ang-1 level was independently associated with MTACcr, while serum Ang-1 level was independently associated with Prcl. Based on the three-pore model of Rippe and colleagues [34], solute and water transport occurs through three types of pores located in the peritoneal capillary endothelium. Lower Ang-1 level, resulting in a disbalanced Ang-1/Ang-2 ratio in favor of Ang-2, might be closely linked to angiogenesis in the local peritoneal cavity. Increased peritoneal vascular surface area, such as angiogenesis, mainly resulting in a larger small pore area might contribute to increased MTACcr. This might be the reason for dialysate Ang-1 level negatively correlating with MTACcr. However, peritoneal protein leakage actually depended on large-pore transport which is much few. Prcl might be more closely correlated with systemic microvascular endothelial layer hyperpermeability, resulted mainly from circulating endothelial integrity impairment and larger inter-endothelial gaps. Recent studies have also shown that systemic Ang-1 could prevent the permeability effects of VEGF by stabilizing the pericyte-endothelial cell association and strengthening inter-endothelial junctions [35, 36]. Thus, decreased serum Ang-1 levels or lower serum Ang-1/Ang2 ratio might be involved in greater peritoneal protein loss.

We found that peritoneal glucose exposure was independently associated with MTACcr. It is well known that long-term success of PD depends mainly on the ability of the peritoneal membrane to provide adequate solute clearances and ultrafiltration. Therefore, with the gradual decline of residual renal function, greater dialysis doses or hypertonic dialysis solution were usually required to maintain the adequate solute removal and euvolemia. However, exposure to hypertonic dialysis solution has long been suspected as a mechanism of peritoneal membrane injury [24]. Glucose was recognized as a strong proinflammatory agent and in a clinical evaluation, Fujimori et al. [37] showed that higher glucose concentration induced higher production of intraperitoneal IL-6. A recent study demonstrated that the use of glucose-based solutions was associated with higher levels of VEGF [38]. Therefore, these studies suggested that glucose could induce local peritoneal inflammation and angiogenesis through direct and indirect effects, consequently resulting in higher MTACcr.

IL-6 has been established to be a central mediator of inflammatory response in the peritoneal cavity and its level increased with therapy duration [39]. Pecoits-Filho et al. [39] found that intraperitoneal and systemic IL-6 and soluble IL-6 receptor (sIL-6R) levels were closely related to peritoneal small-solute transport. However, Lambie et al. [40] showed that local but not systemic inflammation was associated with peritoneal small-solute transport. In present study, we found that dialysate IL-6 levels were independent risk factors contributing to higher MTACcr. Oh et al. [41] recently reported that intraperitoneal IL-6 level was significantly associated with inflammatory and angiogenic factors, such as MCP-1, VEGF and Ang-2. These studies suggested that IL-6 might directly promote or indirectly mediate angiogenesis, which in turn lead to an increased peritoneal vascular surface area and a larger small pore area in the local peritoneal cavity. This might be the reason for intraperitoneal IL-6 levels associated with MTACcr. In addition, we found that dialysate IL-6 level was not an independent risk factor affecting Prcl. It has been reported that peritoneal protein leakage actually depended on peritoneal large-pore transport, which was, for the most part, correlated with systemic endothelial dysfunction [13, 29]. Protein leak across large pores was increased during systemic vascular or by increased hydrostatic pressure across the capillary [42]. Thus, Prcl may serve as a marker for the severity of systemic vascular disease and injury. Clearly further studies are needed to investigate the underlying mechanism.

In parallel with previous reports [39, 43, 44], we found that the dialysate concentrations of IL-6, IL-10 and VEGF were higher than their serum counterparts, respectively. This suggested the local production of these molecules in the peritoneal cavity, reflecting chronic inflammatory state of the peritoneum apart from the systemic inflammation. Unlike the aforementioned molecules analyzed in our study, serum concentrations of Ang-1 and sTie-2 were > 100 times higher than their dialysate concentrations and serum concentration of Ang-2 was almost 20 times higher than its dialysate countpart. So it was reasonable, with such remarkable concentration gradients, that the intraperitoneal concentrations of these molecules were more heavily dependent on their transport from the systemic circulation rather than the local production in the peritoneum.

Our study has several limitations. This was a single center study; however, we have detailed information about patients’ characteristics and laboratory data, and the same doctors and nurses took care of all patients thereby avoiding potential confounders of different centers. Secondly, both incident and prevalent patients were investigated. Thirdly, the study was cross-sectional in nature and the cytokines levels were measured once at enrollment. The effect of time-average cytokines levels might be underestimated. Besides, since there is a lot of possible underlying mechanisms and causative correlations, it is difficult to draw such function disturbances based upon only one measurement of cytokines, and clearly more time depended measurements are required. Further prospective follow-up studies of these patients are needed to elucidate the cause and effect relationships between these associated cytokines and peritoneal transport rate of small molecules and macromolecules.

## Conclusion

Our study indicated that BSA, history glucose exposure, dialysate IL-6 AR and dialysate Ang-1 AR were independent associated factors of MTACcr, while BSA and serum Ang-1 levels were independently associated with Prcl. These results suggested that peritoneal small solute transport and peritoneal large molecular transport might be affected by slightly different panels of inflammatory and angiogenic factors.

## Abbreviations

ACEI/ARB: Angiotensin-Converting Enzyme Inhibitor/Angiotensin Receptor Blocker
Ang-1: Angiopoietin-1
Ang-2: Angiopoietin-2
AR: The dialysate appearance rate
BSA: Body surface area
CAPD: Chronic ambulatory peritoneal dialysis
CVD: Cardiovascular disease
D/P cr: Dialysate/Plasma creatinine ratio
DM: Diabetes mellitus
Kt/V urea: Urea clearance index
MTACcr: Mass transfer area coefficient of creatinine
nPCR,: Normalized protein catabolic rate
PD: Peritoneal dialysis
PET: Peritoneal equilibration test
Prcl: Peritoneal protein clearance
RRF: Residual glomerular filtration rate
sTie-2: Soluble Tie-2
VEGF: Vascular endothelial growth factor
